# Bis(triphenyl­phosphanyl­idene)iminium dichloridotriphenyl­stannate(IV)

**DOI:** 10.1107/S1600536811035422

**Published:** 2011-09-14

**Authors:** Lucio De Lorentiis, Claudia Graiff, Giovanni Predieri

**Affiliations:** a Dipartimento di Chimica GIAF, Viale delle Scienze, 17/A, Università di Parma, 43100 Parma, Italy

## Abstract

The structure of the title compound, [Ph_3_P=N=PPh_3_]^+^[Ph_3_SnCl_2_]^−^ or (C_36_H_30_NP_2_)[Sn(C_6_H_5_)_3_Cl_2_], obtained as a by product of the reaction between Ph_3_SnCl and [Ph_3_P=N=PPh_3_]^+^·HSeO_3_^−^, consists of discrete essentially isolated ions. Both the cation and the anion lie on twofold axes which pass through the central N atom in the cation and through the Sn^IV^ atom in the anion. In the crystal, the ions inter­act only through a weak inter­action between the Cl atom of the anion and an H atom of a phenyl ring of the cation.

## Related literature

For general background to selenite compounds, see: Delferro *et al.* (2010 ▶, 2011 ▶). For related structures, see: Harrison *et al.* (1978 ▶); Nayek *et al.* (2010 ▶); Ng (1995 ▶, 1999 ▶). For details of the Cambridge Crystal Structure Database, see: Allen (2002 ▶).
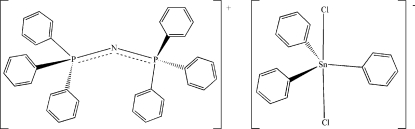


## Experimental

### 

#### Crystal data

(C_36_H_30_NP_2_)[Sn(C_6_H_5_)_3_Cl_2_]
*M* *_r_* = 959.44
Orthorhombic, 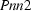

*a* = 17.9119 (6) Å
*b* = 9.7744 (3) Å
*c* = 13.3835 (4) Å
*V* = 2343.16 (13) Å^3^
*Z* = 2
Mo *K*α radiation
μ = 0.76 mm^−1^
*T* = 296 K
0.42 × 0.22 × 0.18 mm

#### Data collection

Bruker APEXII CCD diffractometer
Absorption correction: multi-scan (*SADABS*; Bruker, 2007 ▶) *T*_min_ = 0.629, *T*_max_ = 0.746
36432 measured reflections
7179 independent reflections
6352 reflections with *I* > 2σ(*I*)
*R*_int_ = 0.026

#### Refinement

*R*[*F* ^2^ > 2σ(*F* ^2^)] = 0.025
*wR*(*F* ^2^) = 0.065
*S* = 1.04
7179 reflections
273 parameters
1 restraint
H-atom parameters constrained
Δρ_max_ = 0.20 e Å^−3^
Δρ_min_ = −0.25 e Å^−3^
Absolute structure: Flack (1983 ▶), 3430 Friedel pairs
Flack parameter: −0.022 (13)

### 

Data collection: *APEX2* (Bruker, 2007 ▶); cell refinement: *SAINT* (Bruker, 2007 ▶); data reduction: *SAINT*; program(s) used to solve structure: *SHELXS97* (Sheldrick, 2008 ▶); program(s) used to refine structure: *SHELXL97* (Sheldrick, 2008 ▶); molecular graphics: *ORTEP-3 for Windows* (Farrugia, 1997 ▶); software used to prepare material for publication: *WinGX* (Farrugia, 1999 ▶).

## Supplementary Material

Crystal structure: contains datablock(s) I, global. DOI: 10.1107/S1600536811035422/su2296sup1.cif

Structure factors: contains datablock(s) I. DOI: 10.1107/S1600536811035422/su2296Isup2.hkl

Supplementary material file. DOI: 10.1107/S1600536811035422/su2296Isup3.mol

Additional supplementary materials:  crystallographic information; 3D view; checkCIF report

## Figures and Tables

**Table 1:** Hydrogen-bond geometry (Å, °)

*D*—H⋯*A*	*D*—H	H⋯*A*	*D*⋯*A*	*D*—H⋯*A*
C9—H9⋯Cl1^i^	0.93	2.79	3.718 (2)	173

Symmetry code: (i) 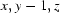.

**Fig. 1.**
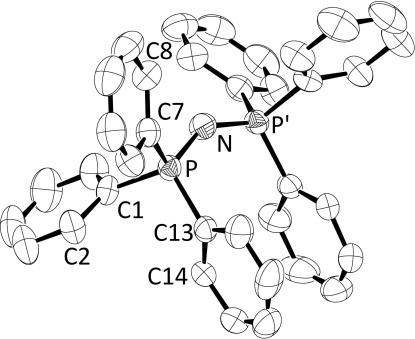
ORTEP drawing of the [Ph3P=N=PPh3]+ cation, showing the atom labelling and the displacement ellipsoids drawn at the 30% probability level. Hydrogen atoms are omitted for clarity. Symmetry code for generating equivalent atoms: ' = -x + 2, -y + 1, z.

**Fig. 2.**
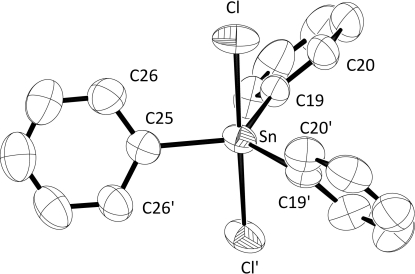
ORTEP drawing of the [Ph3SnCl2]- anion, showing the atom labelling and the displacement ellipsoids drawn at the 30% probability level. Hydrogen atoms are omitted for clarity. Symmetry code for generating equivalent atoms: ' = -x + 2, -y + 2, z.

**Fig. 3.**
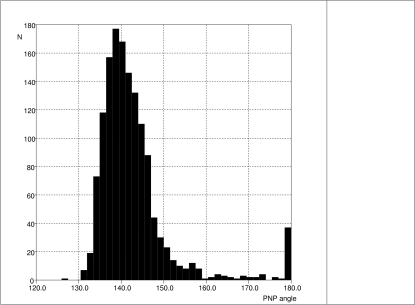
Histogram showing the distribution of the P—N—P bond angle over the 1409 [Ph3P=N=PPh3]+ cations reported in the Cambridge Structural Database (Allen, 2002).

## supplementary crystallographic information

### Comment

The title compound was isolated from a dichloromethane solution as a by product of the reaction between of Ph_3_SnCl with [Ph_3_P=N=PPh_3_]^+^ HSeO_3_^-^ in dichloromethane solvent. The hydrogen selenite salt, prepared in the framework of our research activity on selenite compounds (Delferro *et al.*, 2010, Delferro *et al.*, 2011), contained a significant amount of [Ph_3_P=N=PPh_3_]^+^ Cl^-^, which is responsible of the formation of the title compound.

The structure of the title compound consists of discrete [Ph_3_P=N=PPh_3_]^+^ and [Ph_3_SnCl_2_]^-^ ions (Fig. 1). The [Ph_3_P=N=PPh_3_]^+^, (or PPN^+^ for simplicity), cation is rather typical, lieing on a two fold axis that passes through the central N atom, N1. The P1—N1 bond distance of 1.5763 (9) Å and the P1—N1—P1^i^ [symmetry code (i) = -*x* + 2, -*y* + 1, *z*] bond angle of 141.9 (2)° are in good agreement with the average values of 1.577 (6) Å and 143 (9)° found in 1409 PPN^+^ cations reported in the Cambridge Crystal Structure Database (CSD, V5.32, last update May 2011; Allen, 2002), see Fig. 3. In particular, examining the 1409 PPN^+^ cations it is evident that less than 40 examples present a linear geometry at the nitrogen atom. On excluding these cases the mean value of the P—N—P bond angle is reduced to 142 (7)°, even more in agreement with the angle in the title compound. The P atom is tetrahedral, with the C—P—C and C—P—N angles averaging 109 (3)°.

The [Ph_3_SnCl_2_]^-^ anion exhibits trigonal bipyramidal geometry at the tin atom, with the usual equatorial arrangement of organic groups and the chlorine atoms occupying axial positions. The anion is lying on a two-fold axis passing through the tin atom and atom C25 and C28 of one phenyl ring. The Cl1—Sn1 bond distance is 2.5858 (4) Å and the Cl—Sn—Cl^ii^ [symmetry code: (ii) = -*x* + 2, -*y* + 2, *z*] bond angle is 177.83 (4)°, both in agreement with the values found in four examples (Ng, 1995,1999; Harrison *et al.*, 1978; Nayek *et al.*, 2010) reported in the CSD: The mean values are 2.590 (2) Å and 176.6 (7)°, respectively. The mean planes of the phenyl rings form dihedral angles of 59.70 (2)° and 38.78 (2)° with the SnC_3_ mean plane.

In the crystal there is a weak interaction between the chlorine atom of the dichlorotriphenylstannate anion and a hydrogen atom of a phenyl ring of the bis(triphenylphosphine)iminium cation (Table 1).

### Experimental

A dichloromethane solution of equimolar amounts of triphenyl-tin chloride and bis(triphenylphosphine)iminium hydrogenselenite was stirred at room temperature for 1 h. The solution was then cooled slowly to 278 K. Crystals suitable for X-ray analysis were obtained from the solution in two days.

### Refinement

The C-bound H-atoms were included in calculated positions and treated as riding atoms: C-H = 0.93 Å for CH(aromatic), with U_iso_(H) = 1.2U_eq_(parent C-atom).

### Crystal data

**Table:**

(C_36_H_30_NP_2_)[Sn(C_6_H_5_)_3_Cl_2_]	*F*(000) = 980
*M**_r_* = 959.44	*D*_x_ = 1.360 Mg m^−^^3^
Orthorhombic, *P**n**n*2	Mo *K*α radiation, λ = 0.71073 Å
Hall symbol: P 2 -2n	Cell parameters from 999 reflections
*a* = 17.9119 (6) Å	θ = 3–27°
*b* = 9.7744 (3) Å	µ = 0.76 mm^−^^1^
*c* = 13.3835 (4) Å	*T* = 296 K
*V* = 2343.16 (13) Å^3^	Prism, colourless
*Z* = 2	0.42 × 0.22 × 0.18 mm

### Data collection

**Table:**

Bruker APEXII CCD diffractometer	7179 independent reflections
Radiation source: fine-focus sealed tube	6352 reflections with *I* > 2σ(*I*)
graphite	*R*_int_ = 0.026
ω scans	θ_max_ = 30.6°, θ_min_ = 1.9°
Absorption correction: multi-scan (*SADABS*; Bruker, 2007)	*h* = −25→25
*T*_min_ = 0.629, *T*_max_ = 0.746	*k* = −13→13
36432 measured reflections	*l* = −19→19

### Refinement

**Table:**

Refinement on *F*^2^	Secondary atom site location: difference Fourier map
Least-squares matrix: full	Hydrogen site location: inferred from neighbouring sites
*R*[*F*^2^ > 2σ(*F*^2^)] = 0.025	H-atom parameters constrained
*wR*(*F*^2^) = 0.065	*w* = 1/[σ^2^(*F*_o_^2^) + (0.0339*P*)^2^ + 0.062*P*] where *P* = (*F*_o_^2^ + 2*F*_c_^2^)/3
*S* = 1.04	(Δ/σ)_max_ = 0.001
7179 reflections	Δρ_max_ = 0.20 e Å^−^^3^
273 parameters	Δρ_min_ = −0.25 e Å^−^^3^
1 restraint	Absolute structure: Flack (1983), 3430 Friedel pairs
Primary atom site location: structure-invariant direct methods	Flack parameter: −0.022 (13)

### Special details

**Table:**

Geometry. All e.s.d.'s (except the e.s.d. in the dihedral angle between two l.s. planes) are estimated using the full covariance matrix. The cell e.s.d.'s are taken into account individually in the estimation of e.s.d.'s in distances, angles and torsion angles; correlations between e.s.d.'s in cell parameters are only used when they are defined by crystal symmetry. An approximate (isotropic) treatment of cell e.s.d.'s is used for estimating e.s.d.'s involving l.s. planes.
Refinement. Refinement of *F*^2^ against ALL reflections. The weighted *R*-factor *wR* and goodness of fit *S* are based on *F*^2^, conventional *R*-factors *R* are based on *F*, with *F* set to zero for negative *F*^2^. The threshold expression of *F*^2^ > σ(*F*^2^) is used only for calculating *R*-factors(gt) *etc*. and is not relevant to the choice of reflections for refinement. *R*-factors based on *F*^2^ are statistically about twice as large as those based on *F*, and *R*- factors based on ALL data will be even larger.

### Fractional atomic coordinates and isotropic or equivalent isotropic displacement parameters (Å^2^)

**Table:**

	*x*	*y*	*z*	*U*_iso_*/*U*_eq_	
C1	0.88435 (9)	0.69033 (16)	0.54090 (12)	0.0432 (3)	
C2	0.81206 (10)	0.72511 (17)	0.51192 (17)	0.0576 (5)	
H2	0.7811	0.6596	0.4832	0.069*	
C3	0.78656 (12)	0.8566 (2)	0.5259 (2)	0.0726 (6)	
H3	0.7384	0.8800	0.5065	0.087*	
C4	0.83185 (16)	0.9526 (2)	0.5683 (2)	0.0786 (7)	
H4	0.8146	1.0416	0.5764	0.094*	
C5	0.90228 (15)	0.9193 (2)	0.5988 (2)	0.0812 (7)	
H5	0.9323	0.9852	0.6287	0.097*	
C6	0.92925 (11)	0.7871 (2)	0.58522 (17)	0.0611 (5)	
H6	0.9772	0.7643	0.6059	0.073*	
C7	0.85781 (9)	0.40724 (16)	0.59273 (12)	0.0436 (3)	
C8	0.88264 (11)	0.3604 (2)	0.68448 (15)	0.0542 (4)	
H8	0.9306	0.3818	0.7060	0.065*	
C9	0.83627 (14)	0.2816 (2)	0.74452 (16)	0.0677 (5)	
H9	0.8530	0.2500	0.8061	0.081*	
C10	0.76534 (13)	0.2506 (2)	0.71193 (19)	0.0711 (6)	
H10	0.7339	0.1989	0.7524	0.085*	
C11	0.74047 (11)	0.2948 (2)	0.62107 (19)	0.0660 (6)	
H11	0.6928	0.2713	0.5995	0.079*	
C12	0.78589 (9)	0.37442 (19)	0.56092 (16)	0.0537 (4)	
H12	0.7685	0.4059	0.4996	0.064*	
C13	0.90382 (10)	0.48460 (16)	0.39035 (14)	0.0428 (3)	
C14	0.91498 (10)	0.5907 (2)	0.32243 (14)	0.0507 (4)	
H14	0.9251	0.6784	0.3456	0.061*	
C15	0.91117 (12)	0.5664 (3)	0.22073 (16)	0.0654 (5)	
H15	0.9192	0.6376	0.1758	0.078*	
C16	0.89552 (13)	0.4373 (3)	0.18605 (18)	0.0725 (6)	
H16	0.8922	0.4215	0.1177	0.087*	
C17	0.88481 (14)	0.3321 (3)	0.2517 (2)	0.0783 (7)	
H17	0.8744	0.2450	0.2273	0.094*	
C18	0.88920 (11)	0.3529 (2)	0.35474 (17)	0.0601 (5)	
H18	0.8825	0.2804	0.3989	0.072*	
C19	0.93324 (11)	0.8460 (2)	0.92452 (14)	0.0555 (4)	
C20	0.94493 (15)	0.7072 (2)	0.9444 (2)	0.0787 (6)	
H20	0.9844	0.6807	0.9852	0.094*	
C21	0.89793 (18)	0.6079 (3)	0.9033 (3)	0.0981 (8)	
H21	0.9064	0.5160	0.9172	0.118*	
C22	0.84039 (17)	0.6437 (3)	0.8437 (2)	0.0908 (8)	
H22	0.8097	0.5766	0.8166	0.109*	
C23	0.82711 (15)	0.7791 (3)	0.82299 (19)	0.0835 (7)	
H23	0.7874	0.8035	0.7819	0.100*	
C24	0.87280 (12)	0.8799 (2)	0.86322 (17)	0.0681 (5)	
H24	0.8630	0.9714	0.8492	0.082*	
C25	1.0000	1.0000	1.1555 (2)	0.0497 (6)	
C26	1.06462 (16)	1.0253 (2)	1.20891 (19)	0.0680 (6)	
H26	1.1092	1.0408	1.1752	0.082*	
C27	1.0633 (2)	1.0278 (3)	1.3133 (2)	0.0940 (11)	
H27	1.1066	1.0488	1.3484	0.113*	
C28	1.0000	1.0000	1.3635 (3)	0.104 (2)	
H28	1.0000	1.0000	1.4329	0.125*	
N1	1.0000	0.5000	0.55925 (18)	0.0477 (5)	
P1	0.91740 (2)	0.51807 (4)	0.52080 (3)	0.03734 (9)	
Sn1	1.0000	1.0000	0.996036 (19)	0.04870 (5)	
Cl1	0.88562 (3)	1.16132 (5)	0.99969 (5)	0.07087 (13)	

### Atomic displacement parameters (Å^2^)

**Table:**

	*U*^11^	*U*^22^	*U*^33^	*U*^12^	*U*^13^	*U*^23^
C1	0.0413 (8)	0.0429 (7)	0.0452 (8)	−0.0004 (6)	0.0109 (6)	−0.0061 (6)
C2	0.0472 (8)	0.0522 (8)	0.0735 (14)	0.0078 (6)	0.0013 (9)	−0.0093 (10)
C3	0.0606 (11)	0.0593 (11)	0.0979 (19)	0.0178 (9)	0.0169 (11)	−0.0040 (11)
C4	0.0870 (17)	0.0482 (10)	0.1007 (18)	0.0135 (11)	0.0296 (14)	−0.0106 (11)
C5	0.0852 (16)	0.0531 (12)	0.105 (2)	−0.0121 (11)	0.0140 (14)	−0.0259 (12)
C6	0.0527 (10)	0.0546 (10)	0.0760 (13)	−0.0077 (8)	0.0047 (9)	−0.0150 (9)
C7	0.0381 (7)	0.0415 (7)	0.0513 (9)	−0.0010 (6)	0.0064 (7)	−0.0023 (7)
C8	0.0569 (11)	0.0566 (10)	0.0490 (10)	−0.0034 (8)	0.0032 (8)	−0.0030 (8)
C9	0.0928 (16)	0.0613 (11)	0.0491 (10)	−0.0055 (10)	0.0154 (10)	0.0016 (9)
C10	0.0732 (14)	0.0598 (12)	0.0802 (15)	−0.0155 (10)	0.0313 (12)	−0.0049 (10)
C11	0.0459 (10)	0.0605 (11)	0.0914 (16)	−0.0101 (8)	0.0166 (10)	−0.0056 (11)
C12	0.0390 (8)	0.0516 (10)	0.0703 (12)	−0.0042 (7)	0.0018 (8)	0.0020 (8)
C13	0.0355 (7)	0.0512 (9)	0.0418 (9)	0.0077 (6)	−0.0030 (6)	−0.0088 (6)
C14	0.0497 (9)	0.0579 (9)	0.0445 (9)	0.0199 (7)	−0.0037 (7)	0.0002 (7)
C15	0.0618 (11)	0.0859 (15)	0.0484 (10)	0.0300 (11)	−0.0056 (9)	0.0042 (10)
C16	0.0651 (13)	0.1060 (18)	0.0464 (11)	0.0206 (13)	−0.0085 (9)	−0.0204 (13)
C17	0.0743 (14)	0.0871 (17)	0.0736 (16)	−0.0019 (12)	0.0025 (12)	−0.0441 (14)
C18	0.0597 (11)	0.0558 (11)	0.0648 (13)	−0.0010 (8)	0.0035 (9)	−0.0120 (9)
C19	0.0593 (11)	0.0659 (11)	0.0414 (9)	0.0071 (8)	0.0049 (8)	−0.0087 (8)
C20	0.0872 (16)	0.0715 (13)	0.0774 (15)	0.0129 (12)	−0.0088 (12)	−0.0081 (11)
C21	0.113 (2)	0.0713 (16)	0.110 (2)	−0.0072 (15)	−0.0013 (18)	−0.0146 (16)
C22	0.0943 (19)	0.0950 (19)	0.0831 (18)	−0.0156 (15)	0.0095 (15)	−0.0308 (14)
C23	0.0711 (15)	0.114 (2)	0.0650 (14)	0.0008 (14)	−0.0075 (11)	−0.0255 (14)
C24	0.0700 (13)	0.0775 (13)	0.0567 (11)	0.0032 (10)	−0.0080 (9)	−0.0113 (10)
C25	0.0626 (16)	0.0476 (13)	0.0389 (13)	−0.0052 (10)	0.000	0.000
C26	0.0810 (15)	0.0662 (11)	0.0568 (13)	−0.0148 (10)	−0.0186 (11)	0.0072 (9)
C27	0.152 (3)	0.0699 (14)	0.0604 (16)	−0.0191 (16)	−0.0434 (19)	0.0006 (12)
C28	0.210 (7)	0.064 (2)	0.0396 (17)	−0.012 (2)	0.000	0.000
N1	0.0341 (9)	0.0663 (13)	0.0427 (11)	0.0008 (8)	0.000	0.000
P1	0.03077 (16)	0.04225 (16)	0.0390 (2)	0.00106 (13)	0.00104 (13)	−0.00220 (15)
Sn1	0.04949 (8)	0.06206 (9)	0.03456 (7)	0.00872 (6)	0.000	0.000
Cl1	0.0614 (2)	0.0894 (3)	0.0618 (3)	0.0292 (2)	−0.0110 (3)	−0.0201 (3)

### Geometric parameters (Å, °)

**Table:**

C1—C6	1.376 (2)	C16—C17	1.367 (4)
C1—C2	1.394 (2)	C16—H16	0.9300
C1—P1	1.8049 (16)	C17—C18	1.396 (4)
C2—C3	1.377 (2)	C17—H17	0.9300
C2—H2	0.9300	C18—H18	0.9300
C3—C4	1.364 (4)	C19—C24	1.398 (3)
C3—H3	0.9300	C19—C20	1.398 (3)
C4—C5	1.365 (4)	C19—Sn1	2.1476 (19)
C4—H4	0.9300	C20—C21	1.397 (4)
C5—C6	1.391 (3)	C20—H20	0.9300
C5—H5	0.9300	C21—C22	1.349 (4)
C6—H6	0.9300	C21—H21	0.9300
C7—C8	1.384 (3)	C22—C23	1.373 (4)
C7—C12	1.394 (2)	C22—H22	0.9300
C7—P1	1.7998 (16)	C23—C24	1.389 (3)
C8—C9	1.389 (3)	C23—H23	0.9300
C8—H8	0.9300	C24—H24	0.9300
C9—C10	1.377 (3)	C25—C26^i^	1.383 (3)
C9—H9	0.9300	C25—C26	1.383 (3)
C10—C11	1.365 (4)	C25—Sn1	2.134 (3)
C10—H10	0.9300	C26—C27	1.397 (4)
C11—C12	1.384 (3)	C26—H26	0.9300
C11—H11	0.9300	C27—C28	1.346 (5)
C12—H12	0.9300	C27—H27	0.9300
C13—C18	1.398 (3)	C28—C27^i^	1.345 (5)
C13—C14	1.393 (3)	C28—H28	0.9300
C13—P1	1.7929 (19)	N1—P1^ii^	1.5763 (9)
C14—C15	1.383 (3)	N1—P1	1.5763 (9)
C14—H14	0.9300	Sn1—C19^i^	2.1476 (19)
C15—C16	1.374 (4)	Sn1—Cl1	2.5858 (4)
C15—H15	0.9300	Sn1—Cl1^i^	2.5858 (4)
			
C6—C1—C2	119.70 (16)	C17—C18—C13	118.8 (2)
C6—C1—P1	120.91 (14)	C17—C18—H18	120.6
C2—C1—P1	119.39 (12)	C13—C18—H18	120.6
C1—C2—C3	119.87 (18)	C24—C19—C20	117.2 (2)
C1—C2—H2	120.1	C24—C19—Sn1	121.74 (16)
C3—C2—H2	120.1	C20—C19—Sn1	120.81 (16)
C4—C3—C2	120.1 (2)	C21—C20—C19	120.6 (3)
C4—C3—H3	120.0	C21—C20—H20	119.7
C2—C3—H3	120.0	C19—C20—H20	119.7
C5—C4—C3	120.7 (2)	C22—C21—C20	120.8 (3)
C5—C4—H4	119.7	C22—C21—H21	119.6
C3—C4—H4	119.7	C20—C21—H21	119.6
C4—C5—C6	120.2 (2)	C23—C22—C21	120.1 (3)
C4—C5—H5	119.9	C23—C22—H22	119.9
C6—C5—H5	119.9	C21—C22—H22	119.9
C1—C6—C5	119.4 (2)	C22—C23—C24	120.2 (3)
C1—C6—H6	120.3	C22—C23—H23	119.9
C5—C6—H6	120.3	C24—C23—H23	119.9
C8—C7—C12	119.46 (16)	C19—C24—C23	121.0 (2)
C8—C7—P1	118.89 (13)	C19—C24—H24	119.5
C12—C7—P1	121.54 (14)	C23—C24—H24	119.5
C7—C8—C9	120.3 (2)	C26^i^—C25—C26	117.7 (3)
C7—C8—H8	119.8	C26^i^—C25—Sn1	121.14 (16)
C9—C8—H8	119.8	C26—C25—Sn1	121.14 (16)
C8—C9—C10	119.4 (2)	C25—C26—C27	120.4 (3)
C8—C9—H9	120.3	C25—C26—H26	119.8
C10—C9—H9	120.3	C27—C26—H26	119.8
C11—C10—C9	120.91 (19)	C28—C27—C26	120.6 (3)
C11—C10—H10	119.5	C28—C27—H27	119.7
C9—C10—H10	119.5	C26—C27—H27	119.7
C10—C11—C12	120.3 (2)	C27^i^—C28—C27	120.1 (4)
C10—C11—H11	119.9	C27^i^—C28—H28	119.9
C12—C11—H11	119.9	C27—C28—H28	119.9
C11—C12—C7	119.66 (19)	P1^ii^—N1—P1	141.90 (17)
C11—C12—H12	120.2	N1—P1—C13	115.13 (10)
C7—C12—H12	120.2	N1—P1—C7	108.34 (8)
C18—C13—C14	119.33 (18)	C13—P1—C7	109.30 (8)
C18—C13—P1	121.71 (15)	N1—P1—C1	111.33 (6)
C14—C13—P1	118.69 (13)	C13—P1—C1	105.72 (8)
C15—C14—C13	120.5 (2)	C7—P1—C1	106.69 (7)
C15—C14—H14	119.8	C25—Sn1—C19^i^	116.46 (5)
C13—C14—H14	119.8	C25—Sn1—C19	116.46 (5)
C16—C15—C14	120.0 (2)	C19^i^—Sn1—C19	127.07 (10)
C16—C15—H15	120.0	C25—Sn1—Cl1	88.916 (18)
C14—C15—H15	120.0	C19^i^—Sn1—Cl1	91.27 (5)
C17—C16—C15	120.2 (2)	C19—Sn1—Cl1	89.70 (5)
C17—C16—H16	119.9	C25—Sn1—Cl1^i^	88.917 (18)
C15—C16—H16	119.9	C19^i^—Sn1—Cl1^i^	89.70 (5)
C16—C17—C18	121.2 (2)	C19—Sn1—Cl1^i^	91.27 (5)
C16—C17—H17	119.4	Cl1—Sn1—Cl1^i^	177.83 (4)
C18—C17—H17	119.4		

Symmetry codes: (i) −*x*+2, −*y*+2, *z*; (ii) −*x*+2, −*y*+1, *z*.

### Hydrogen-bond geometry (Å, °)

**Table:**

*D*—H···*A*	*D*—H	H···*A*	*D*···*A*	*D*—H···*A*
C9—H9···Cl1^iii^	0.93	2.79	3.718 (2)	173

Symmetry codes: (iii) *x*, *y*−1, *z*.
